# Morphological and Biochemical Changes in Rat Hippocampal Tissue Following Exposure to Different Doses of Cisplatin

**DOI:** 10.3390/brainsci16070740

**Published:** 2026-07-13

**Authors:** Milorad Antić, Vladimir Antić, Dušan Sokolović

**Affiliations:** 1 Department of Anatomy, Faculty of Medicine, University of Niš, 18000 Niš, Serbia; antic.miki87@gmail.com; 2 Faculty of Sports and Physical Education, University of Niš, 18000 Niš, Serbia; vlada.antic@hotmail.com; 3 Department of Biochemistry, Faculty of Medicine, University of Niš, 18000 Niš, Serbia; 4 Department of Laboratory Diagnostics, Institute for Treatment and Rehabilitation “Niška Banja”, 18000 Niš, Serbia

**Keywords:** cisplatin, hippocampus, oxidative damage, apoptosis, morphological changes

## Abstract

**Highlights:**

**What are the main findings?**

- Single-dose cisplatin induced dose-dependent oxidative stress, inflammation, apoptosis, and structural alterations in rat hippocampal tissue.
- Cisplatin at 10 mg/kg produced the most pronounced hippocampal neuronal degeneration and increase in caspase-3 content.

**What are the implications of the main findings?**

- Acute cisplatin exposure can trigger significant hippocampal injury within five days, supporting the role of oxidative and inflammatory pathways.
- A single dose of 10 mg/kg represents a suitable experimental model for investigating mechanisms of cisplatin-induced hippocampal damage and potential neuroprotective interventions.

**Abstract:**

*Background*/*Objectives*: Cisplatin (CP) is a platinum-based chemotherapeutic agent associated with neurotoxicity and cognitive impairment. Due to its high metabolic activity and sensitivity to oxidative stress, the hippocampus represents a particularly vulnerable brain structure. The present study evaluated dose-dependent hippocampal alterations following single-dose cisplatin administration in rats using biochemical, histopathological, and morphometric analyses. *Methods*: Male Wistar rats were divided into four groups (n = 8): control and cisplatin-treated groups receiving single intraperitoneal doses of 8, 9, or 10 mg/kg. Five days after treatment, hippocampal tissue was analyzed for oxidative stress and inflammatory, apoptotic, and morphological changes. *Results*: Cisplatin administration significantly increased TBARS and AOPP levels, indicating enhanced lipid and protein oxidation. Elevated hippocampal TNF-α, IL-6, and IL-1β levels demonstrated activation of inflammatory pathways, particularly in animals receiving 9 and 10 mg/kg cisplatin. Higher doses additionally increased Bax/Bcl-2 ratio, caspase-3 content, and DNase I/II activity, consistent with enhanced apoptotic signaling. Histopathological examination revealed neuronal degeneration, pyknotic nuclei, pericellular halo formation, and progressive disruption of hippocampal cytoarchitecture, while morphometric analysis demonstrated significant alterations in neuronal nuclear surface area, predominantly in the 10 mg/kg group. *Conclusions*: These findings demonstrate that acute cisplatin exposure induces oxidative, inflammatory, apoptotic, and structural progressive hippocampal injury with a threshold-like increase in several endpoints at higher cisplatin doses, with 10 mg/kg producing the most pronounced neurotoxic effects.

## 1. Introduction

The hippocampus is a complex subcortical structure located in the medial temporal lobe and represents a key component of the limbic system, with a central role in the formation and consolidation of long-term memory, particularly episodic and autobiographical memory [1]. Morphologically, it has a characteristic S-shaped curvature, is composed of densely packed neuronal layers, and is anatomically divided into the Cornu ammonis, comprising four regions (CA1–CA4), and the dentate gyrus (DG) [2]. The dentate gyrus contains granule cells and is involved in the formation of new memory traces and novelty detection, whereas the CA regions, comprised mainly of pyramidal neurons, play a crucial role in synaptic plasticity and long-term potentiation as the basis of memory encoding [2]. As mentioned, the hippocampus can be divided into several distinct regions based on differences in cellular morphology and function [2]. The DG contains the smallest and most densely packed neuronal cell bodies, whereas the CA4 region is characterized by large, loosely distributed polymorphic neurons situated between the two arms of the DG. In Ammon’s horn, the CA3 field contains relatively large pyramidal neurons arranged in a compact layer, CA2 forms a narrow transition zone with similarly large but more loosely organized pyramidal cells, and CA1 is composed of smaller pyramidal neurons that gradually become less densely packed toward the subiculum [3]. This structure is known to be very sensitive to different damaging agents, both exogenous and endogenous, that can rapidly cause changes in neural cell structure and function [4]. These changes in the hippocampal structure and function can be seen in the foundation of numerous psychiatric and neurological disorders [5]. 

Cisplatin (CP) is a platinum-based cytostatic (chemotherapeutic), used for the treatment of solid tumors (testicular, ovarian, lung cancers, etc.) [6]. Its mechanism of action is based on intracellular activation following diffusion into the cell, where, in a low-chloride environment, it undergoes aquation and forms reactive complexes capable of interacting with nucleophilic molecules, particularly DNA, leading to the formation of DNA adducts and intrastrand cross-links [6,7]. These DNA lesions disrupt replication and transcription processes, resulting in cell cycle arrest and induction of cell death via apoptosis or necrosis, depending on cisplatin concentration and the metabolic state of the cell [6]. In addition to DNA damage, cisplatin also interacts with proteins, membrane phospholipids, and antioxidant systems, contributing to oxidative stress and further enhancing its cytotoxic effects [6,8]. Also, CP is known to cause inflammatory changes in the central nervous system, affecting nerve impulse transmission and cell functioning [6]. Most importantly, significant concentrations of CP can be found in the brain tissue of both animals and humans exposed to this drug [9].

There are different models examining the effect of cisplatin on the central nervous system and the extent of changes that it produces. These rodent models are defined by differences in dosing and administration regimens, ranging from acute single-dose exposure (doses from 6 to 10 mg/kg, in some extreme cases even 20 mg/kg) [10,11] to repetitive administration protocols (from 5 to 8 mg/kg per cycle over multiple injections) [10,11,12], yielding somewhat contradictory results. In this light, chronic and repeated application does produce significant and pronounced changes in different brain structures, affecting various cascades associated with inflammation and cell death [10]. However, acute exposure has been suggested to cause rapid changes in both nerve cells and microglia [12], while other researchers have suggested the need for a prolonged exposure to CP in order to reach such an effect [10,13]. Also, there is uncertainty as to whether the exposure to CP causes an inflammatory response in the central nervous system or not, and whether this effect is dose-dependent [12].

Considering these issues, the aim of the current study was to evaluate the extent of hippocampal changes associated with different doses of cisplatin in rats exposed to a single dose of CP. This was to be achieved by examining the extent of oxidative damage and apoptosis in the hippocampal tissue of affected rats. Finally, the measured biochemical changes would be corroborated with pathohistological and morphometrical ones.

## 2. Materials and Methods

### 2.1. Drugs and Chemicals

Cisplatin was obtained from TEVA (Actavis d.o.o., Leskovac, Serbia) in the form of a solution (50 mg/100 mL) and administered intraperitoneally (i.p.) to experimental animals. All other chemicals and reagents used in the study were of analytical grade and purchased from Sigma-Aldrich (St. Louis, MO, USA) or Carl Roth (Karlsruhe, Germany).

### 2.2. Animals and Housing

Male Wistar rats (200–250 g, approximately 7 weeks old) were used in this experiment. Animals were housed under standard laboratory conditions (22 ± 2 °C, 12 h light/dark cycle) with free access to food and water. Prior to the experiment, all animals were allowed to acclimatize for one week. All experimental procedures were conducted in accordance with European Union Directive 2010/63/EU and approved by the Ethics Committee of the Republic of Serbia (323-07-01762/2019-05 obtained on 1 March 2019).

### 2.3. Experimental Design

The animals were randomly divided (using a random number generator) into four groups (n = 8 per group): one control group and three experimental groups, with the doses selected based on previous studies [14,15] and some pilot experiments. The selected doses were chosen to encompass the upper range of acute cisplatin exposure most frequently used in experimental neurotoxicity studies. Preliminary pilot studies were also conducted to confirm the feasibility of the experimental protocol under our laboratory conditions. Based on previous reports and our pilot observations, this dose interval was expected to capture the transition from mild biochemical alterations to pronounced hippocampal injury while avoiding excessive systemic toxicity associated with higher doses. The control group received an equivalent volume of vehicle, while experimental groups were treated with cisplatin administered as a single intraperitoneal injection. Cisplatin was applied in three different doses: 8 mg/kg, 9 mg/kg, and 10 mg/kg. Five days later the treatment animals were sacrificed by overdose of ketamine (injected i.p. in a dose of 200 mg/kg) following the approved guidelines of the local Ethics committee. Blood samples were collected by cardiac puncture, and brain tissue was carefully dissected for further analyses. 

### 2.4. Tissue Isolation and Processing

Immediately after sacrifice, the brain was rapidly removed and placed on an ice-cold surface, after which it was measured. The hippocampus was carefully dissected from a random hemisphere according to anatomical landmarks, avoiding contamination with surrounding cortical tissue. Isolated hippocampal samples were briefly rinsed in cold saline, blotted dry, and weighed. Hippocampal tissue was homogenized, on ice, in an appropriate volume of ice-cold PBS buffer (1:10, *w*/*v*) using a mechanical homogenizer. The obtained homogenates were then centrifuged for 15 min at 12,000 rpm at 4 °C, and the supernatants were collected and stored (−80 °C) for further biochemical analyses. Tissue protein content was determined following a previously described procedure which is based on the Folin–Ciocalteu method and relies on the colorimetric reaction between protein-bound copper ions and the Folin–Ciocalteu reagent [16]. For histopathological evaluation after isolation of the brain, a contralateral brain hemisphere was washed and fixed in 10% buffered formalin. Biochemical and histopathological evaluations were performed by investigators blinded to the treatment protocol and animal grouping.

### 2.5. Biochemical Analysis

#### 2.5.1. Oxidative Damage Marker Measurement

The quantification of thiobarbituric acid reactive substances (TBARS) was performed using a standard method as previously described [17]. Briefly, 100 µL of hippocampal tissue homogenate was heated at 95 °C in the presence of thiobarbituric acid solution until the formation of a chromogenic product. The absorbance of the colored reaction mixture was measured at 540 nm using a Multiscan Ascent spectrophotometer (Labsystems, Vantaa, Finland). The concentration of TBARS in each sample was calculated using a standard curve generated with 1,1,3,3-tetraethoxypropane as the TBARS equivalent, and the levels were expressed as nanomoles per mg of protein.

Advanced oxidation protein products (AOPPs) in hippocampal tissue samples were determined spectrophotometrically using the previously described method [18]. The assay is based on a colorimetric reaction between oxidized proteins and chlorinated oxidants. Briefly, the samples were diluted 1:10 in phosphate-buffered saline (PBS), followed by the addition of 15 μL of potassium iodide to each well of a microtiter plate. Subsequently, 30 μL of glacial acetic acid was added to all wells. Absorbance was measured at 340 nm, and the absorbance of the sample blank was subtracted from that of the corresponding sample wells. Chloramine-T equivalent concentrations of AOPPs were normalized to total protein content and expressed as μmol/mg protein.

#### 2.5.2. Inflammatory Cytokines Measurement

The levels of interleukin-6 (IL-6), interleukin-1β (IL-1β), and tumor necrosis factor-α (TNF-α) were measured in hippocampal supernatant using ELISA kits Abcam (Cambridge, UK), in accordance with the manufacturers’ instructions.

#### 2.5.3. Determination of BAX and BCL-2 Protein Levels

The levels of BAX and Bcl-2 proteins in hippocampal homogenates were determined using commercially available ELISA kits (Rat BAX (Bcl-2 Associated X Protein) (E-EL-R0098) and Rat Bcl-2 (B-cell Lymphoma/Leukemia 2) (E-EL-R0096), Elabscience Biotechnology Inc., Wuhan, Hubei, China). Briefly, homogenate samples (100 μL) were added to microplate wells pre-coated with antibodies specific for rat Bax or Bcl-2 and incubated according to the manufacturer’s protocol. After washing steps, enzyme-conjugated detection antibodies were applied. Following the incubation period and wash step, the color substrate was added to each well. The reaction was terminated using stop solution and the absorbance of the reaction mixture was measured at 450 nm. The content was calculated based on the standard curve with range from 0.16 to 10 ng/mL and the final concentrations were expressed as ng/mg of proteins. The Bax/Bcl-2 ratio was used as an indicator of apoptotic signaling.

#### 2.5.4. Determination of Caspase-3 Levels

Caspase-3 levels in hippocampal homogenates were quantified using rat-specific ELISA kit Elabscience Biotechnology Co., Ltd. (E-EL-R0160; Wuhan, Hubei, China), in accordance with the manufacturers’ instructions.

#### 2.5.5. Determination of DNase I and DNase II Activity

For DNase I activity, samples were incubated in reaction buffer containing 50 mM Tris-HCl, 10 mM MgCl_2_, and 1 mM CaCl_2_, pH 7.5. For DNase II activity, homogenates were incubated in 50 mM sodium acetate buffer, pH 5.0. Calf thymus DNA in both experiments was used as substrate. Reactions were incubated at 37 °C for 30–60 min and stopped with EDTA. The DNA degradation was measured at 260 nm and expressed relative to protein content [19].

### 2.6. Histological Analysis

The isolated brain tissue samples fixed in 10% neutral buffered formalin were further processed through graded alcohols and embedded in paraffin. Brain tissue sections (4–5 µm) were subsequently stained with hematoxylin and eosin (H&E) according to standard protocol. Changes in the hippocampal cells were examined and described. Histopathological alterations in hippocampal tissue were evaluated semiquantitatively based on the degree of neuronal degeneration, presence of pyknotic neurons, pericellular halo formation, and disruption of normal cytoarchitecture using a grading scale ranging from absent (−), (+) mild (++), moderate to severe (+++). Scores were obtained from the hippocampal tissue of 8 animals per group by a single observer (M.A.), unaware of the treatment, examining at least 3 sections per animal. Morphometric analysis was performed on 10 randomly selected images, obtained at 400× magnification, using ImageJ (version 1.54p) analysis software [20,21]. The hippocampal regions CA1, CA2, CA3, CA4 and the dentate gyrus (DG) were identified based on their characteristic cytoarchitectonic features. At least 50 µm^2^ nuclear surface area per region were measured for each specimen. For morphometric analysis, at least 50 neuronal nuclei were measured per hippocampal region in each specimen. To avoid pseudoreplication, individual nuclei were not treated as independent statistical units. Instead, the mean nuclear surface area was first calculated for each hippocampal region within each animal, and these per-animal mean values were used for group-level statistical comparisons (n = 8 animals per group). The obtained results are expressed as mean values.

### 2.7. Statistical Analysis

Results were expressed as the mean ± SD. Prior to statistical analysis, data distribution was assessed using the Shapiro–Wilk test, and homogeneity of variance was evaluated using Levene’s test. Statistically significant differences were determined by one-way analysis of variance (ANOVA) followed by Tukey’s post hoc test for multiple comparison (GraphPad Prism version 5.03, San Diego, CA, USA). Probability values (*p*) less than 0.05 were considered to be statistically significant.

## 3. Results

### 3.1. Changes in Brain Tissue Weight

Brain mass did not differ significantly among the experimental groups (*p* > 0.05). In the control group, brain mass was 1.85 ± 0.04 g, while values observed in CP-treated groups were: 1.84 ± 0.05 g (8 mg/kg), 1.82 ± 0.04 g (9 mg/kg), and 1.80 ± 0.05 g (10 mg/kg). Although the mean values showed a numerical decrease in brain mass with increase in cisplatin dose, these differences were not statistically significant (*p* > 0.05).

### 3.2. Influence of Different Doses of CP on Hippocampal Oxidative Stress

**Figure 1:**
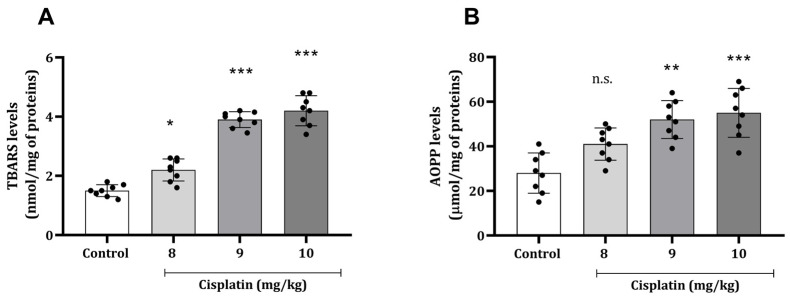
The effects of different doses of cisplatin on oxidative stress were estimated through TBARS (**A**) and AOPP (**B**) levels in rat hippocampal tissue. Data are given as mean ± SD, n = 8. ANOVA followed by Tukey’s post hoc test, * *p* < 0.05, ** *p* < 0.01, *** *p* < 0.001 vs. control, n.s.—no significant difference.

Application of CP in different doses led to enhanced lipid peroxidation, measured through an increase in TBARS levels, and protein oxidation, measured through an increase in AOPP levels (Figure 1). In the case of TBARS, statistically significant elevation was observed in the group of animals that received 8 mg/kg of CP compared with the control group, and in those that received 9 and 10 mg/kg of CP as well (Figure 1A). In the case of AOPP, only two higher doses of CP (9 and 10 mg/kg) produced a significant increase in the studied parameter (Figure 1B).

### 3.3. Impact of CP on Inflammatory Cytokine Levels in Hippocampus

Exposure of rats to a single dose of CP led to a significant increase in hippocampal proinflammatory cytokine levels, which became evident predominantly at 9 and 10 mg/kg (Table 1). The significant effect on TNF-α and IL-6 levels was observed in rats exposed to 9 and 10 mg/kg of CP, while the significant effect on IL-1β levels was demonstrated with all applied doses of CP (Table 1). Compared with control levels, a single dose of 10 mg/kg CP produced a dose-dependent increase in hippocampal cytokine levels, with TNF-α increasing up to 3.2-fold, IL-6 up to 3-fold, and IL-1β up to 3.5-fold.

### 3.4. Apoptosis-Associated Parameters Evaluation

**Figure 2:**
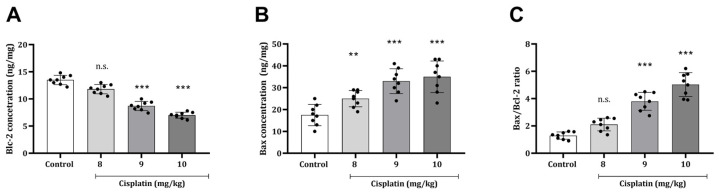
Changes in Bcl-2 (**A**) and Bax (**B**) content, and Bax/Bcl-2 ratio (**C**) in the hippocampi of rats belonging to different groups. Data are presented as mean value ± SD (n = 8). ANOVA followed by Tukey’s post hoc test, *** *p* < 0.001, ** *p* < 0.01 vs. control group, n.s.—no significant difference.

In the present experimental setting, application of cisplatin in a dose of 8 mg/kg did not produce any notable changes, compared to the control, in the hippocampal Bcl-2 and Bax content (Figure 2A,B). On the other hand, application of cisplatin in a dose of 9 and 10 mg/kg produced a statistically significant decrease in Bcl-2 (Figure 2A) and an increase in Bax (Figure 2B) content, followed by an increase in Bax/Bcl-2 ratio (Figure 2C).

**Figure 3:**
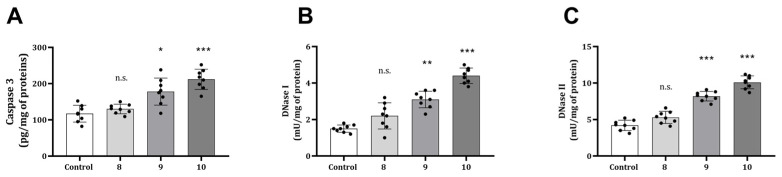
Caspase-3 content (**A**), and alkaline (**B**) and acidic (**C**) DNaase activity in hippocampus tissue obtained from rats belonging to different experimental groups. Data are presented as mean value ± SD (n = 8). ANOVA followed by Tukey’s post hoc test, *** *p* < 0.001, ** *p* < 0.01, * *p* < 0.05 vs. control group, n.s.—no significant difference.

The progressive increase in caspase-3 levels in rats receiving different doses of CP is shown in Figure 3A, with statistically significantly elevated values observed in rats treated with 9 and 10 mg/kg, when compared with the control group. Similarly, DNase I and DNase II activity increase became statistical significance in rats that received 9 and 10 mg/kg (Figure 3B,C). 

### 3.5. Histopathological and Morphometric Analysis of Hippocampal Tissue of the Treated Rats

**Figure 4:**
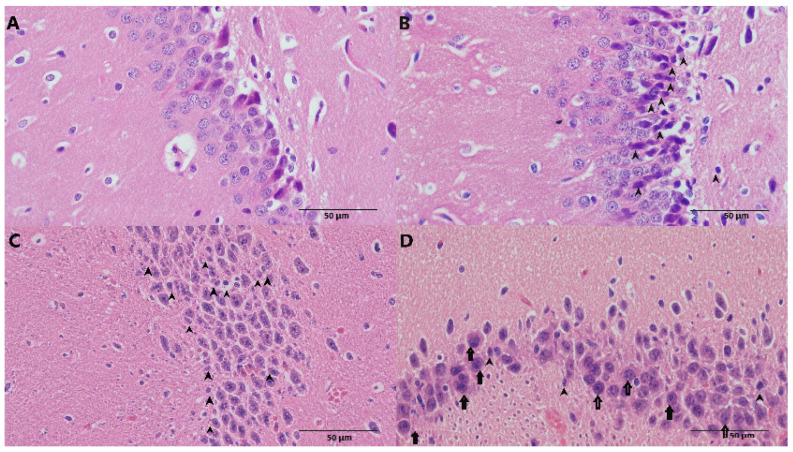
(**A**) Hippocampal sections demonstrating preserved cytoarchitecture with intact pyramidal neuronal organization of DG. ((**B**)—CA1 region) and ((**C**)—CA1 region) mild neuronal injury with slight disorganization of the pyramidal layer, occasional neuronal shrinkage (noted with arrowhead), and minimal degenerative alterations in animals receiving 8 and 9 mg/kg of CP, respectively. (**D**) Pronounced alterations with marked neuronal degeneration (noted with arrow), cytoarchitectural disorganization, extensive pericellular clearing, and increased number of pyknotic neurons in CA3 region.

Hippocampal cells in the control group appeared normal and, without any pathological substrate and only around occasional cells, a pericellular halo can be seen (Figure 4A, Table 2). With increasing concentration of the applied CP, an increase in the extent of changes in the hippocampal CA and DG cells could be observed (Figure 4B–D, Table 2). In lower doses, 8 and 9 mg/kg, moderate neuronal degeneration with cytoplasmic shrinkage, pyknotic nuclei, pericellular halo formation, and focal disruption of normal cytoarchitecture was visible (Figure 4B,C, Table 2). In animals with the highest applied dose of CP (10 mg/kg), degeneration in the hippocampal pyramidal layer, including pyknotic and shrunken neurons, vacuolar changes, and disruption of normal cytoarchitecture through the examined tissue was present.

Morphometric analysis of the nuclear areas in the hippocampal tissue of rats exposed to different concentrations of CP showed them to be altered compared to the control group parameters. Significant alterations in nuclear areas were found only in the group of animals exposed to the highest dose of CP (10 mg/kg) in CA1, CA2 and CA3, but not in CA4 and DG, compared to the control group (Table 3). There were no significant differences between the nuclear surface areas in any of the hippocampal areas in groups that received 8 and 9 mg/kg of CP, when compared to the control (Table 3).

## 4. Discussion

For some time now, it has been recognized that cisplatin induces neurotoxicity accompanied by structural alterations in the central nervous system [6]. While previous experimental and clinical studies have reported structural brain changes following cisplatin exposure [22,23,24], no significant differences in total brain mass were observed in the present study. This finding should be interpreted with caution, as whole-brain wet weight is a relatively crude measure that is unlikely to detect subtle or region-specific alterations, particularly after acute exposure. Therefore, the absence of changes in total brain mass does not preclude the pronounced biochemical, histopathological, and morphometric alterations observed in the hippocampus.

Although oxidative stress, neuroinflammation, apoptosis, and brain injury have previously been described following cisplatin administration, considerable heterogeneity remains among experimental models with respect to dose, treatment schedule, and evaluated endpoints. Consequently, direct comparison between studies is difficult, particularly regarding the temporal sequence of biochemical and structural alterations after acute cisplatin exposure. The present study was designed to address this gap by applying the most relevant and most frequently used acute cisplatin doses within a single standardized experimental model and evaluating oxidative stress, inflammatory response, apoptotic signaling, histopathological alterations, and morphometric changes simultaneously. This integrated approach enabled direct comparison of molecular and structural alterations across doses and provided a more comprehensive characterization of dose-specific hippocampal neurotoxicity. Although the selected CP doses do not directly correspond to clinical dosing regimens, they represent an established acute experimental model for investigating cisplatin-induced neurotoxicity.

As mentioned, the hippocampi, or to be more precise, nerve cells located within them, are highly sensitive to hypoxia, since it leads to a reduction in ATP production and alteration in cell membrane potential [25]. During a time of impaired mitochondrial function and decreased ATP production, cells build up toxic oxygen products, free radicals, that through direct reaction with cell structures cause damage to lipids and proteins [25]. The present study shows how 5 days post-exposure to CP is sufficient for a certain amount of lipid and protein oxidatively modified molecules, estimated through TBARS and AOPP, to accumulate in hippocampal tissue (Figure 1A,B). Generated oxygen radicals interact with polyunsaturated fatty acids, part of cell membranes, forming TBARS that directly alter membrane fluidity and further pushing cells towards apoptosis and necrosis [26]. The excess oxygen radicals also directly oxidizes the amino acid residues of proteins, promoting the formation of carbonyls (AOPP) and causing protein cross-linkage, which leads to their functional impairment and structural degradation (e.g., enzymes and receptors) [15]. The most modest changes were observed in rats treated with 8 mg/kg of CP, while more pronounced ones were observed in those treated with 9 and 10 mg/kg. The altered molecules and the entire initiated oxidative cascade are a powerful stimulus for both inflammation and cell death [27]. The time required to initiate and propagate oxidative stress-related responses is debatable and varies based on the results of the studies. Interestingly, in an acute experiment, a dose of 8 mg/kg of cisplatin 24 h after the injection produced significant alterations in whole brain MDA content (up to 35% increase from the control values), suggesting that shortly after the application, there are observable changes in brain tissue, possibly caused by affecting antioxidative enzymes [28]. Interestingly, studies rarely examine whether there are significant morphological changes that follow biochemical ones shortly after exposure.

One of the mechanisms underlying CP neurotoxicity is NF-kB-mediated inflammatory response through an upregulation of pro-inflammatory cytokines and chemokines [29,30] initiated via different mechanisms. In the present study, the three most prominent cytokines, IL-6, IL-1β, and TNF-α, which are frequently found in connection with chemotherapy-associated brain damage [29], were measured and found to be significantly increased in rats exposed to CP (Table 1). TNF-α is a prototypical inflammatory cytokine involved in the pathophysiology of CP-induced damage to different organs and cells, and is known to activate other pro-inflammatory cytokines [31]. On the other hand, increased IL-1β production has been linked with potential microglia activation, which further leads to nerve cell death [32]. The role of IL-6 is potentially also related to cognitive decline, since the levels of this cytokine are found to be increased in the cerebrospinal fluid, blood and hippocampal tissue of patients with Alzheimer’s disease [33], and it is believed to be especially important for CP neurotoxicity [34]. In an experimental setting involving repeated exposure to a low dose of CP (5 mg/kg/week), a significant increase in the mentioned inflammatory cytokines was noted [31,32]. In another study, repeated application of CP in a dose of 8 mg/kg over 8 days produced a significant increase in the mentioned parameters [35]. An increase in the observed parameters with a dose of CP this low can potentially be explained by repeated exposure rather than a more acute effect, which is expected in the present experimental setting. 

Oxidative stress, cell damage and inflammation are known to be closely related to the activation of apoptosis [36]. As discussed and proven by the results of this study, there are observable changes in the oxidative damage parameters and inflammation markers (Figure 1 and Table 1), which could influence the apoptotic process. Also, one of the mechanisms that might be associated with hippocampal dysfunction and damage involves the formation of DNA cross-links [37], a known mode of action of cisplatin [6], which further activates cell death. Cell function alterations and signaling cascades push the cell to events resulting in an increase in pro-apoptotic proteins (e.g., Bax–associated with DNA damage) and their translocation into mitochondria, resulting in cytochrome C leakage. At the same time, anti-apoptotic proteins (e.g., Bcl-2) serve to prevent mitochondrial content leakage and thus prevent further downstream cascade activation [36]. Previous research indicates that CP administration in a dose of 7.5 mg/kg results in a shift toward pro-apoptotic signaling in rat prefrontal cortex tissue, which is characterized by increased Bax and decreased Bcl-2 relative gene expression, consequently increasing the Bax/Bcl-2 ratio [38]. Also, immunohistochemically quantified Bcl-2 and Bax ratio in both cerebral cortex and hippocampus was found to be increased in animals receiving a single dose (5, 7.5 and 12 mg/kg) of cisplatin. The lowest dose did not produce any significant increase in the Bax/Bcl-2 ratio, while the two higher doses, 7.5 and 12 mg/kg, significantly increased cerebral cortex and hippocampal Bax/Bcl-2 ratio [39]. These findings indicate that apoptotic signaling becomes pronounced only beyond a certain dose threshold, with higher doses producing a stronger shift toward pro-apoptotic balance. The results of the present study indicate that the dose of 8 mg/kg of CP does not cause significant alterations in the Bax/Bcl-2 ratio (Figure 2C), although it produces changes in oxidative stress parameters (Figure 1) and inflammatory parameters (Table 1). On the other hand, the two higher doses, 9 and 10 mg/kg, do produce significant changes in the two studied proteins associated with the apoptosis process, which were accompanied by alterations in oxidative stress and inflammatory markers (Figure 1 and Table 1). 

Initiation of apoptosis is finally conducted via execution effector cysteine protease, caspase-3, known for cleaving multiple substrates, after being activated, leading the cell to programmed destruction. Activated caspase-3 is known to promote apoptotic DNA fragmentation, primarily through cleavage of the inhibitor of caspase-activated DNase (ICAD/DFF45), thereby releasing caspase-activated DNase (CAD/DFF40) [36]. Multiple doses of CP, as low as 2 [40] and 5 mg/kg [34] during a prolonged application period, increase the caspase-3 in the hippocampus. Interestingly, a single dose of 10 mg/kg induces an increase in caspase-3 activity in the lungs of the treated rats, just 5 days after treatment [41]. Here, the obtained results showed that a significant increase in caspase-3 is only visible with doses 9 and 10 mg/kg (Figure 3A). As previously mentioned, activated caspase-3 indirectly further activates DNase via CAD, which, to the best of our knowledge, has not been examined in the hippocampal tissue of rats treated with CP. In some previous studies, a single dose of CP (10 mg/kg) has been found to activate DNase activity in the lung [41], while a dose of 8 mg/kg induces an increase in DNase activity in the kidneys [42] days after application. Thus, apart from direct DNA damage induced by CP [6], the observed effects on DNA and the alterations in the nucleus shape (Table 3) might arise from the activity of the DNases. Therefore, the present findings extend the current understanding of CP-induced neurotoxicity by suggesting that DNase activation may represent an additional downstream mechanism associated with DNA degradation and nuclear remodeling during hippocampal neuronal apoptosis.

An additional contribution of the present study is the simultaneous evaluation of multiple stages of CP-induced neuronal injury. Oxidative stress and inflammation were detectable following lower CP doses, while significant activation of apoptotic pathways together with measurable morphometric alterations occurred predominantly after higher dose application. These findings suggest that oxidative and inflammatory changes precede overt structural injury, providing further insight into the sequence of pathological events during acute cisplatin-induced hippocampal neurotoxicity.

On top of biochemical changes during the process of apoptosis, the cell and nucleus undergo different morphological changes depending on the state of the process [43]. The alterations in the nucleus, reflecting initial phases of apoptosis [43], have been studied here through the changes in nucleus shape descriptors of hippocampal cells in animals receiving different doses of CP (Table 3). Activation of effector caspases induces apoptosis, which is characterized by morphological changes, including cell shrinkage, chromatin condensation, nuclear fragmentation, plasma membrane alterations, and the formation of apoptotic bodies [44], which are partially observed in the study’s sample (Figure 4B–D, Table 3). Previous studies showed that CP at a dose of 8 mg/kg cause significant changes in cell morphology in the CA3 region and DG after a single injection, associating these results with the glutamatergic neurotransmission [28]. The results of the present research do not comply with these findings, showing that the dose of 8 mg/kg does not alter significantly neuron nucleus appearance in any of the hippocampal fields (Table 3). In the present experimental setting, the observable changes in the CA1 and CA2 regions were only visible with the highest applied dose (10 mg/kg). Much higher doses of CP (20 mg/kg), 3 days after the injection, caused clear and marked changes in hippocampal dendritic morphology [11]. The morphometric findings should be interpreted with caution because changes in neuronal nuclear surface area were not uniform across hippocampal regions. The reduction in nuclear area observed in CA1 and the DG is consistent with neuronal shrinkage, chromatin condensation, and pyknotic changes described in the histopathological assessment. In contrast, the increased nuclear area observed in CA2 and CA3 does not necessarily indicate apoptosis and may reflect early degenerative swelling, edematous/reactive cellular changes, or region-specific vulnerability to acute cisplatin exposure. Therefore, the morphometric data should be interpreted as evidence of heterogeneous hippocampal structural injury rather than as a uniform apoptotic response. Additional analyses using cell-specific markers and apoptosis-specific staining would be required to determine whether enlarged nuclei represent reactive neuronal changes, glial responses, or non-apoptotic forms of cellular injury.

The present study has several limitations that should be acknowledged. First, behavioral assessments were not performed; therefore, although the observed hippocampal biochemical, apoptotic, histopathological, and morphometric alterations are consistent with mechanisms implicated in cognitive dysfunction, no direct conclusions regarding cognitive impairment can be drawn. Second, the study focused on a relatively narrow range of acute cisplatin doses, selected to characterize the threshold of hippocampal neurotoxicity rather than establish a comprehensive dose–response relationship across a broader concentration range. Thus, the relatively narrow dose interval represents a deliberate methodological choice rather than a full limitation of the study. Third, the present study used only male rats and the present findings cannot be directly extrapolated to female animals. Another limitation is that Bax, Bcl-2, caspase-3, and inflammatory cytokines were quantified using ELISA only, without orthogonal validation by Western blotting or immunohistochemistry. Future studies including both sexes are warranted to determine whether the molecular, histopathological, and morphometric responses to acute cisplatin exposure differ according to sex. Finally, although oxidative stress, inflammatory mediators, apoptotic markers, and DNase I/II activity were evaluated, the study did not investigate upstream intracellular signaling pathways or perform mechanistic interventions capable of confirming causal relationships, although the investigation of some parameters (DNase I and DNase II) extends the current understanding of molecular events associated with cisplatin-induced neuronal injury.

## 5. Conclusions

The present study demonstrated that acute cisplatin administration, known to produce different forms of organ damage, induced selective alterations in the examined hippocampal parameters, with the most pronounced biochemical and structural changes observed at higher doses. The observed increase in reactive oxygen species, oxidative damage markers, and inflammatory mediators suggests that enhanced oxidative stress may contribute to apoptosis and morphological cell injury in hippocampal tissue. Acute cisplatin-induced hippocampal injury was observed, characterized by threshold-like progression of molecular and structural alterations, with the most significant changes occurring at doses of 9 mg/kg and above. The highest dose (10 mg/kg) produced the most pronounced hippocampal alterations 5 days after administration, indicating that it reliably induces acute hippocampal injury in this experimental model. Importantly, the novelty of the present study lies in the integrated assessment of oxidative, inflammatory, apoptotic, histopathological, and morphometric alterations across different acute cisplatin doses, allowing a more precise characterization of dose-specific hippocampal neurotoxicity. The findings suggest that oxidative and inflammatory responses precede overt structural injury and highlight DNase I/II activation as a potentially important contributor to cisplatin-induced neuronal damage. Future studies should investigate additional molecular mechanisms and clinically relevant experimental paradigms to further elucidate cisplatin-induced hippocampal neurotoxicity and identify potential neuroprotective strategies.

## Figures and Tables

**Table 1:** Hippocampal tissue cytokine levels in rats of different experimental groups.

Parameter	Control	8 mg/kg	9 mg/kg	10 mg/kg
TNF-α (ng/mg)	3.8 ± 0.7	5.5 ± 1.9 ^n.s.^	8.7 ± 2.2 ***	12.0 ± 1.8 ***
IL-6 (ng/mg)	2 ± 0.9	3.5 ± 0.8 ^n.s.^	4.6 ± 1.3 **	6 ± 2.4 ***
IL-1β (ng/mg)	4.1 ± 0.3	8.9 ± 1.2 ***	10.9 ± 0.8 ***	14.5 ± 1.1 ***

Data are mean ± SD, n = 8. ANOVA followed by Tukey’s post hoc test, ** *p* < 0.01, *** *p* < 0.001 vs. control, ^n.s.^—no significant difference.

**Table 2:** Semiquantitative changes in the hippocampal tissue of rats belonging to different groups.

Parameter	Control	8 mg/kg	9 mg/kg	10 mg/kg
Neuronal degeneration	-	+	++	+++
Pyknotic neurons	-	+/-	+	++
Cytoarchitectural disruption	-	+/-	+	++
Pericellular halo	-	+/-	+	++

Scores were obtained from the hippocampal tissue of 8 animals per group.

**Table 3:** Changes in hippocampal neuron nucleus surface area (μm^2^) in different experimental groups.

Region	Control	8 mg/kg	9 mg/kg	10 mg/kg
CA1	59.9 ± 1.5	45.4 ± 5.7 ^n.s.^	44.6 ± 6.1 ^n.s.^	43.1 ± 6.5 ***
CA2	48.1 ± 3.4	63.7 ± 8.9 ^n.s.^	65.3 ± 10.4 ^n.s.^	67.9 ± 7.8 ***
CA3	60.3 ± 1.3	71.1 ± 5.2 ^n.s.^	72.6 ± 5.3 ^n.s.^	73.2 ± 6.1 **
CA4	78.8 ± 1.3	77.6 ± 4.4 ^n.s.^	77.0 ± 5.3 ^n.s.^	76.8 ± 5.6 ^n.s.^
DG	48.0 ± 1.0	39.7 ± 5.6 ^n.s.^	38.6 ± 6.2 ^n.s.^	38.4 ± 6.8 ^n.s.^

Values are presented as mean ± SD calculated from per-animal regional mean values; n = 8 animals per group. Individual nuclear measurements were averaged within each animal before statistical analysis. ANOVA followed by Tukey’s post hoc test. ** *p* < 0.01, *** *p* < 0.001 vs. control, ^n.s.^—no significant difference.

## Data Availability

Data is available upon reasonable request.

## Abbreviations

The following abbreviations are used in this manuscript:

**Abbreviation**	**Full term**
AOPP	Advanced Oxidation Protein Products
ATP	Adenosine Triphosphate
Bax	Bcl-2-associated X protein
Bcl-2	B-cell lymphoma 2
CA1–CA4	Cornu Ammonis regions 1–4
CNS	Central Nervous System
CP	Cisplatin
DG	Dentate Gyrus
DNase	Deoxyribonuclease
ELISA	Enzyme-Linked Immunosorbent Assay
H&E	Hematoxylin and Eosin
HPA	Hypothalamic–Pituitary–Adrenal
i.p.	Intraperitoneal
IL-1β	Interleukin-1 beta
IL-6	Interleukin-6
MDA	Malondialdehyde
NF-κB	Nuclear Factor kappa B
PBS	Phosphate-Buffered Saline
ROS	Reactive Oxygen Species
SD	Standard Deviation
TBARS	Thiobarbituric Acid Reactive Substances
TNF-α	Tumor Necrosis Factor alpha
